# Gilt Management for Fertility and Longevity

**DOI:** 10.3390/ani9070434

**Published:** 2019-07-09

**Authors:** Jennifer Patterson, George Foxcroft

**Affiliations:** AFNS, University of Alberta, Edmonton, AB T6G 2P5, Canada

**Keywords:** gilt development, puberty, sow lifetime productivity, litter of origin

## Abstract

**Simple Summary:**

Improving sow lifetime productivity, herd stability, and maximizing lifetime performance and longevity in the sow herd, represent significant challenges to the swine industry. Routine implementation of efficient gilt development unit (GDU) programs which deliver high quality, breeding-eligible gilts to the sow farm is still needed. Good gilt management starts at birth, because litter of origin, lactation management and the application of early selection strategies are early indicators of future performance and efficiency. A failure to select gilts with the greatest reproductive potential and inappropriate management of their physiological state and metabolic condition at service, are key risk factors for poor sow lifetime productivity (SLP). Management practices that deliver gilts with the greatest potential SLP are crucial to the productivity of conventional production systems.

**Abstract:**

Substantial evidence supports successful management of gilts as an absolutely necessary component of breeding herd management and the pivotal starting point for the future fertility and longevity of the breeding herd. Therefore, gilt management practices from birth have the potential to influence the future reproductive performance of the sow herd. A good gilt management program will address several key components such as birth traits that determine the efficiency of replacement gilt production; effective selection of the most fertile gilts for entry to the breeding herd; effective management programs that provide a consistent supply of service eligible gilts; and appropriate management of weight, physiological maturity, and a positive metabolic state at breeding. Good gilt management can largely resolve the existing gap between excellent genetic potential and the more modest sow lifetime productivity typically achieved in the industry. Investment in good gilt development programs from birth represents a foundational opportunity for improving the efficiency of the pork production industry.

## 1. Introduction

Sow lifetime productivity (SLP) is a complex trait that is influenced by both sow productivity (quality pigs weaned per sow per year) and longevity [1,2,3,4]. Numerous factors impact SLP, including sow fertility and prolificacy, preweaning mortality, nutrition, management, housing and environment, health and stockmanship, and retention in the breeding herd [2,5]. Gilts are the foundation of efficient breeding herd performance [6] and the successful introduction of high-quality, breeding eligible gilts into the breeding herd is often under-estimated as an important driver of SLP [7,8]. Acknowledgement of the outstanding SLP achieved by the top 10% of breeding herds worldwide indicates the true reproductive potential of contemporary commercial dam lines, yet many herds fail to realize this potential. The primary goal of this review is to provide convincing evidence that good gilt management can largely resolve the existing gap between excellent genetic potential and the more modest sow lifetime productivity typically achieved in the industry. Nikkila et al. [9] concluded that “reproductive and feet/leg soundness or locomotion related removal frequencies imply that genetic improvements in both reproductive and structural soundness traits as well as good reproductive management practices are needed to improve SLP”. However, we suggest that in the case of early herd removals for poor reproductive performance, the key issue is inappropriate management of replacement gilts between birth and entry to the breeding herd. Therefore, this review focuses on those aspects of gilt and sow management that relate to key physiological traits that underpin excellent reproductive performance, providing an evidenced-based approach to support proposed management interventions. This approach does not preclude the need to consider many other traits that are part of the integrated selection strategies that support a competitive pork industry, such as selection for conformational traits, robustness, and disease tolerance. Rather, we choose to focus on traits that are affected by management decisions made within the lifetime of the breeding female, irrespective of the specific commercial genotype involved. 

Our underlying belief is that gilt management practices from birth have the potential to influence the future reproductive performance of the sow herd [10]. A good gilt management program will address several key components, including birth traits that determine the efficiency of replacement gilt production, effective selection of the most fertile gilts for entry to the breeding herd, effective management programs that provide a consistent supply of service eligible gilts and appropriate management of weight, physiological maturity, and a positive metabolic state at breeding. Implementation of a breeding management program that recognizes the important link between effective gilt management and excellent SLP is both achievable and cost effective.

## 2. Birth Traits that Determine the Efficiency of Replacement Gilt Production

In recent years, both individual birth weight and litter birth weight phenotype, as well as the sex ratio of the birth litter, have been reported to be predictive factors of future gilt performance.

### 2.1. Low Individual Birth Weight

As a consequence of genetic selection for increased litter size, the industry has seen an associated increase in variation in birth weight within the litter and an increase in the proportion of piglets with low birth weight [11]. Within-litter variation has been attributed to factors such as the duration of ovulation, oocyte maturation, the implantation capability of conceptuses and position within the uterus, placental efficiency, uterine space, breed differences and intrauterine growth retardation [12]. There is consensus that low birth weight gilts have increased preweaning mortality [13,14] and those low birth weight gilts that do survive past the nursery phase have poor growth until finishing and are significantly lighter than their higher birth weight litter mates [13,15]. Additionally, as future replacement females, low birth weight negatively impacts their reproductive potential. Variation in birth weight is negatively correlated to ovarian and uterine development [16] and low birth weight gilts have different populations of follicles on the ovary and shorter vaginal length at 150 days of age, suggesting ovulation rate and consequently litter size may be adversely affected [15]. Collectively, the reports of Valet et al. [17] and Calderon-Diaz et al. [18] suggested that, in high health environments, the birth weight and overall growth rate of contemporary commercial gilts are not limiting for age at puberty, but preweaning growth rate was inversely related to age at puberty and birth weight was positively associated with uterine weight. Similarly, Magnabosco et al. [19] reported that there was no effect of birth weight on the percentage of gilts that showed estrus within 30 days of starting boar exposure at 170 days, nor on age at puberty. However, low birth weight gilts had a higher rate of removal due to anestrus before first mating and gilts weighing <1.0 kg at birth produced fewer total pigs born alive at first farrowing and fewer total pigs over three parities. Taken together, these results show that low birth weight in replacement gilts compromises future growth, production, and longevity.

Post-farrowing management (day one care) is critical to improving the retention and performance of replacement gilts. The main causes of neonatal piglet mortality are chilling, starvation, and crushing by the sow and most preweaning mortality occurs within three days after birth [20]. Low birth weight pigs are compromised, as they generally have lower energy reserves, poorer thermoregulatory abilities, lower vitality, and a decreased ability to acquire colostrum because they are weakened and are less competitive during lactation [21,22,23]. Adequate colostrum intake plays an important role in promoting newborn pig health, growth, and survivability and the effects on subsequent reproductive performance have been well documented [17,22,24,25,26]. From a growth standpoint, colostrum intake and birth weight are positively associated with weaning weight, and higher colostrum intake is more beneficial to pigs with a lower body weight than a higher body weight [17,24,26]. Furthermore, for low birth weight pigs, weaning weight and finishing weight are significantly improved if piglets consume the maximum as compared with the minimum amount of colostrum [21]. From a reproductive standpoint, a low blood immunocrit (an objective measure of immunoglobulin intake) at day one was associated with reduced growth, increased age at puberty, reduced numbers born alive, and reduced preweaning growth rate [17]. This is consistent with previous results from Bartol [25] suggesting that insufficient colostrum ingestion at birth may impair uterine gland development and reproductive performance. Therefore, it may be beneficial to implement strategic cross-fostering strategies on all future replacement females as suggested by Vallet et al. [17] to improve the amount of colostrum ingested by neonatal piglets and, consequently, preweaning growth rates which would be beneficial for future replacement females. 

A reduction in the size of the litter in which replacement females are raised is another management technique that may increase overall growth, enhance early development of reproductive organs, and thus increase longevity and performance. In earlier studies, Nelson and Robinson [27] showed that gilts reared in small litters (six piglets) were heavier at weaning and 140 days of age, and that ovulation rates were improved in their first parity as compared with gilts reared in large litters (14 piglets). Similarly, Deligeorgis et al. [16] reported that preweaning growth rate was impacted when gilts were raised in litters of six, nine or 12 piglets. Using more contemporary commercial dam lines, Flowers [28] reported that gilts raised in litters of less than seven reached puberty earlier, had improved farrowing rates, and better retention over six parities as compared with gilts raised in litters that are greater than 10. In a more recent and commercially relevant study, Flowers [29] used inherent variability in normal farrowing and lactation management to create two neonatal environments for litters born to sows with an established low birth weight phenotype (see below). On average, gilts in the reduced group were born in litters of 14 but nursed in litters of 13, whereas gilts in the normal group were born in litters of 14.5 but nursed in litters of 16. Confirming the earlier results involving much smaller litter sizes, replacement gilts from the reduced litters were heavier at birth and weaning, grew faster during lactation, and had greater lifetime productivity measured as total pigs produced and retention rates by parity three as compared with their counterparts from the normal group.

### 2.2. Low Litter Birth Weight Phenotype

A low litter birth weight phenotype (BWP) is hypothesized to carry all the same risks described above for individual low birth weight gilts but as a “litter” trait. This trait is repeatable over consecutive parities and arises from the interactions between a high ovulation rate, the dynamics of early embryonic survival, and limited placental development early in gestation, irrespective of litter size at term [30,31,32]. Consequently, later in gestation, a repeatable low litter BWP is associated with characteristics of intrauterine growth retardation which negatively affects birth weight, body composition, post-natal survival, growth performance, and testicular development in male pigs [31]. Furthermore, gilts born to a sow with a low BWP have lower retention in the herd within four days of birth, at weaning, and at preselection into the breeding herd [33]. 

The ability to predict a sow’s litter BWP is important and has considerable ramifications for the efficiency of replacement gilt production and the lifetime productivity of gilts produced. Identifying sows that repeatedly display the low BWP also allows producers to selectively apply the relevant management interventions discussed earlier. In the most extreme low BWP population (bottom 15%) at production herd level, Smit et al. [31] reported that no sow first giving birth to a low birth weight litter produced a high birth weight litter at any subsequent farrowing. Therefore, producers can effectively select against extreme low BWP sows without risking missing out on high quality litters born in later parities, and thereby minimize the number of extreme low BWP sows in the nucleus/multiplication herd. This will increase the efficiency of replacement gilt production and also reduce the risk of passing this unfavorable low birth weight trait to the downstream commercial units. 

### 2.3. Sex Ratio

The sex ratio of the litter where the replacement female was born may affect lifetime performance and behavior and could potentially be used as another selection tool at birth [34,35]. Gilts born to litters with a high proportion of males are exposed to increased levels of androgens from their male littermates in utero causing gilts to become masculinized [34,36]. It is generally reported that gilts born in female-biased litters are potentially better replacement females than gilts from male-biased litters, however, more research in this area is needed [34]. When sex ratio was recorded at birth, Lamberson et al. [37] reported that as the proportion of males in the litter at birth increased, age at puberty decreased. Conversely, Drickamer et al. [38] reported that females from litters with a male-biased sex ratio attained puberty later. Nevertheless, further studies reported that gilts from male-biased litters were more likely to have lower successful inseminations, higher insemination failures, lower mating success, fewer pigs born, and less teats as compared with gilts from female-biased litters [35,36,38,39]. Masculinized females from male-biased litters are also more likely to display male-like behaviors, are less likely to be fearful, and more likely to be aggressive than gilts from female-biased litters [40]. Aggression may lead to early removal from the herd, reduced sow productive lifetime [41], and could have important welfare implications [38].

Anogenital distance can be used as an indicator of female masculinization in pigs [36,38]. Drickamer et al. [38] reported that gilts originating from a male-biased litter (>67% males) had a larger anogenital distance when measured within four days after birth as compared with gilts from litters with lower proportions of males in the litter. In contrast, Seyfeng et al. [36] reported that although anogenital distance was not different between male-biased (>60% males) as compared with female-biased (>60% females) litters at day one of age, gilts from female-biased litters had a longer anogenital distance at three and 16 weeks of age. In a second study by the same authors, anogenital distance was measured at the time of preselection at approximately 170 days of age. Gilts with an anogenital distance longer than 11.55 mm, and likely from a female-biased litter, were heavier, achieved puberty earlier, were mated younger, and had greater born alive litter size at parity one than gilts with an anogenital distance shorter than 11.55 mm [36].

Taken together, these results confirm that the sex ratio of the litter into which a potential replacement gilt is born, and the anogenital distance at selection, could also be considered when selecting future replacement females. Although more research is needed in the area, future replacement gilts could be non-selected based on sex ratio at birth, or measurements of anogenital distance cold be taken at the time of selection, to further help in improving the productivity of the replacement female.

## 3. Effective Selection of the Most Fertile Gilts for Entry to the Breeding Herd

Successful gilt introduction and selection drives lifetime reproductive performance and longevity in the breeding herd. Litter size has been reported to increase until the fourth parity and then slowly declines [42]. The failure of females to produce at least three [43,44], or even five [45], litters represents a potential financial loss to the producer and is a major concern for the swine industry. The frequency of culling females from the herd is highest in gilts (38.5–51.1%) [46,47] and a high incidence of sows only producing one litter has been reported [44,48]. Thus, a key area for improvement is from gilt entry until farrowing the third litter, and particularly improved management to reduce the number of gilts that never farrow a litter and are completely unproductive [49]. The ability to identify gilts with the greatest potential for lifetime performance, therefore, is crucial to the productivity of conventional production systems and the response to boar stimulation effectively identifies the more productive gilts. When boar exposure is limited to a pre-established window of time, earlier maturing gilts are identified and producers can take advantage of the link between early sexual maturity and improved sow lifetime productivity [50].

Although “age at puberty” is a reliable indicator of future sow reproductive performance and longevity [3], it is important to recognize that the recorded age at pubertal estrus is an interaction involving genetic potential, the underlying physiological mechanisms affecting sexual maturation and the management protocols implemented. Age at puberty is characterized by a moderate heritability, reportedly ranging between 0.25–0.42 [46]. The variation in farm and management conditions can negatively impact future performance and attention should focus on management to optimize future performance and longevity [51]. Suboptimal management and environment inputs frequently override the underlying genetic potential for early sexual maturation [52]. 

The characteristics of behavioral estrus at puberty are predictive of future performance: Sows with stronger behavioral symptoms during pubertal estrus (length and strength of the standing reflex) are more likely to farrow [53], and gilts with more prominent vulval changes at pubertal estrus also have more prominent vulval changes at their first post-weaning estrus [54]. Early age at puberty was reported to have little effect on the total pigs born or born alive per litter on a per parity basis, or on total pigs produced over the female’s productive life [46,47,54,55]. However, the likelihood of a gilt farrowing a first, second or third litter increased as age at puberty decreased [51,56] and age at puberty is generally associated with improved retention rate and longevity of sows in the herd [46,51,55,57]. Gilts that are younger at puberty are culled at higher parities than gilts that are older at puberty [47,54]. The primary reason for culling of gilts from the herd has been reported to be reproductive failure [48] and for those gilts culled due to reproductive reasons, a higher proportion removed had delayed puberty [47]. Gilts with an early age at puberty are served earlier and thus accumulate fewer lifetime nonproductive days, increasing their lifetime productivity measured as pigs weaned/sow/year [2,46]. Gilts with a lower age at first mating (<229 d) had greater longevity as measured by herd productive days and parity at removal [58]. Additionally, an increase in age at first mating from 220 to 300 days was associated with an increase in culling risk due to pregnancy failure by 2.1% [59]. Furthermore, the lifetime performance of gilts with increased age at first mating is confounded by the fact that these gilts are also at risk of being overweight at breeding [47] which negatively affects longevity. Age at first mating, therefore, is intrinsically related to the biological variation in age at puberty and to herd management [58] and has been shown to be a critical factor determining future longevity and lifetime efficiency [46,58,59,60,61]. Taken together, these results indicate that the detection and recording of pubertal estrus by approximately 220 days of age is a key driver of future reproductive performance [56].

Nevertheless, compared to the detailed analysis of sow reproductive performance as a measure of commercial success, objective and critical monitoring of gilt development, and a clear understanding of the link between the quality of the replacement gilt program and overall breeding herd performance, are lacking. Patterson et al. [55] classified gilts that reached puberty within 40 days of initial boar contact starting at 140 days as “select” and gilts that did not respond within 40 days as “non-select” females. More “select” gilts were initially bred and the rate of fallout per parity tended to be lower as compared with “non-select” females. In general, gilts that are naturally cyclic within a defined number of days after boar exposure (35 to 40 in a commercial situation) should be considered to be the premium “select” gilt population. All others are considered “opportunity” gilts and are only entered into the herd if breeding targets cannot be met from the “premium” select pool. 

The ability to identify early puberty, and to produce a synchronous pubertal response to external stimuli, are both dependent on the age at the start of puberty stimulation and heat detection. When boar exposure commences earlier (140 to 160 d of age), a normal distribution in age at detected first estrus is observed in the majority of the population [56,62,63]. When gilts continue to be stimulated and monitored for longer periods (up to 260 days of age), most will eventually have a recorded estrus, however, the later maturing gilts were reported to be part of a different distribution [63]. Although delaying the start of puberty stimulation to greater than 190 d results in a more synchronous response to boar stimulation [64], this limits the ability to discriminate between the earlier maturing “select” gilts and the later maturing “opportunity” gilts that are less fertile [55]. Therefore, although retention of nonpubertal gilts within the gilt pool for long periods will result in high selection rates, this is probably a counterproductive approach and comes at a cost. Gilts that take longer to respond to boar exposure have longer entry to service intervals and accumulate excessive non-productive days [55], and the management of these later maturing gilts involves inefficient use of labor and space. Most importantly, these later maturing gilts also have reduced retention in the breeding herd [46,47,54,56], have poorer reproductive efficiency over their productive life [54], and are at risk for increased mature body size at breeding, which is associated with poor retention in the breeding herd [63]. 

As reviewed by Knox et al. [65], despite effective puberty stimulation and estrus detection programs being in place, delayed puberty and anestrus are observed are observed on commercial breeding farms, with 10–30% of gilts failing to display estrus within 60 to 80 days of boar exposure. Similarly, Patterson et al. [49] reported that in successive groups of gilts entering the same gilt development unit, between 12% and 43% were noncyclic after 30 days of intensive boar exposure. This variation in response to boar stimuli could be due to a number of factors, including age, growth rate, season, health status, barn environment, crowding, and unknown litter of origin effects. In the case of gilts that failed to display estrus in recorded studies, examination of the ovaries at slaughter indicated that approximately 40% to 60% of the gilts did have inactive ovaries and were truly prepubertal. However, the remainder had active corpora lutea indicating that the gilts had previously cycled but had not been detected in estrus [66,67]. Similarly, in a study by Tummaruk and Kesdangsakonwut [68], in gilts that were culled but at slaughter were confirmed to be pubertal and to have previously ovulated normally, approximately a third were culled because they did not exhibit a standing pubertal estrus. These authors suggest that either ineffective estrus detection or silent heat may be the cause [66,67,68]. For those gilts that were previously cyclic and may have displayed silent heat, Knox [12] suggested this may be due to an underdeveloped hypothalamic-pituitary axis that is unable to mount a positive feedback response to low concentrations of circulating estrogen. However, evidence that gilts cycled and displayed a standing estrus that was undetected at the farm, highlights the importance of implementing effective gilt management programs. 

The ability to correctly diagnose reproductive failure in the gilt is confounded by the complex interactions involved in the stimulation and detection of pubertal estrus [12]. Puberty attainment in gilts can be affected by numerous factors including housing, climatic environment, season, manure handling systems, feeding systems, nutrition programs, health, and numerous management factors [69]. Knox [12] recently identified litter of origin, birth weight, growth rate, and body composition as key factors that may affect pubertal onset in gilts. As previously discussed, gilt management starts at birth and factors such as birth weight, colostrum ingestion, and preweaning growth, if not properly controlled, may delay age at puberty. Despite all these complex interactions, effective pre-selection programs at approximately 150–170 days of age, followed by rigorous and well managed selection protocols identifying and recording a pubertal standing heat, are critical steps for the achievement of good breeding herd performance. 

Kirkwood and Aherne [70] predicted that neither gilt age nor body weight are reliable indices of reproductive development, and this is supported by the large range of age (130–190 d) and growth rate (0.4–0.8 kg/d) at which gilts reached puberty reported by Patterson et al. [55]. However, minimum growth thresholds appear necessary. Beltranena et al. [71] suggested that at growth rates below 0.55 kg/d the onset of puberty may be delayed. More recently, the negative relationship between age and lifetime growth rate at puberty has been confirmed [50,62,72]. Given the growth rates achieved in contemporary dam-line genotypes, few gilts are at risk of low growth rates (>0.55 kg/d), however, puberty onset will still be delayed in slower growing gilts [63]. Conversely, in high health and high productivity herds, ~40% of gilts are achieving growth rates >0.70 kg/d and are at risk of growing too fast if feed intake is not limited during development. This is a major concern for those sectors of the pork industry that practice feeding to appetite throughout gilt development. Calderón Díaz et al. [63] reported that overweight gilts at breeding are at risk for reduced SLP and are a risk factor for early culling from the sow herd. To reduce the risk of the fastest growing gilts being overweight at breeding, reducing the age at the start of boar exposure and thus identifying puberty sooner, may enable producers to breed gilts earlier and lighter. Kummer et al. [72] reported no adverse effects on reproductive performance over three parities in breeding gilts growing >700 g/d at their second estrus, and a minimum of 127 kg, when breeding occurred between 185 and 210 d of age, as compared with >210 d of age. 

## 4. Effective Management Programs that Provide a Consistent Supply of Service Eligible Gilts

Developing management practices that identify gilts with the greatest potential for lifetime performance is crucial to the productivity of conventional production systems [50,55,58]. Therefore, the implementation of an effective gilt development system is the pivotal starting point to select gilts with the greatest reproductive potential. 

### 4.1. The Boar Effect

The boar is a critical factor influencing puberty attainment in gilts and daily exposures to a rotation of mature, high libido, boars maximizes the response to boar exposure. The boar effect is a combination of tactile, visual, auditory, and olfactory cues [73]. Olfactory cues have been identified as being the most important and “priming” boar pheromones identified in saliva act through nasal receptors and the olfactory bulb to induce pubertal estrus in gilts [74]. “White-type” boars most commonly used in commercial production should be a minimum of 10 months of age to ensure that they are secreting adequate levels of the “primer” pheromones and the salivary “froth” that incorporates an essential binding protein for these steroids [69]. Even when using a purpose-designed boar exposure area (BEAR) for stimulating pubertal estrus [75]), direct contact with a boar reduces age at puberty and increases the percentage of gilts cycling, as compared with fence-line contact [69,76]. Furthermore, Patterson et al. [77] reported that taking the gilts to the BEAR is more effective in inducing puberty than taking the same boars to group-housed gilts in pens. This is consistent with the findings of Rekowt et al. [78] that even after the boar’s removal from his pen, the remaining pheromones may be sufficient to induce early puberty. Boar libido is also an important factor, gilts exposed to high libido boars reached puberty nearly nine days earlier than gilts exposed to low libido boars [79]. To maintain libido, it is recommended that boars are routinely permitted to mount a gilt in standing estrus and to be “hand collected” [50]. Daily, direct exposure to a rotation of mature boars for a minimum of 10 to 15 min per day maximizes the response to this “priming” component of the “boar effect” [69,80]. Therefore, a planned boar replacement program that provides a consistent supply of quality boars for puberty stimulation is an essential component of a gilt puberty stimulation program. 

### 4.2. Implementation of an Effective Puberty Stimulation Program

To maximize these components of the “boar effect”, and to efficiently, effectively, and safely stimulate first puberty and identify the most fertile gilts, a purpose-designed puberty stimulation area is invaluable [75]. Implementation of an effective gilt development unit (GDU) program in conjunction with the use of a BEAR facility has been shown to identify earlier maturing gilts, and thus to take advantage of the link between early sexual maturity and improved SLP [50,55,77]. A BEAR system facilitates both the stimulation and detection of puberty by providing both fence-line and direct contact (15 min daily) with multiple mature boars [50]. The GDU protocol is divided into two periods, comprising pre-stimulation management followed by an aggressive but limited stimulation program. During pre-stimulation, routine procedures such as vaccinations, sorting, and tagging are completed, as these procedures may disrupt feed intake and have negative consequences on puberty induction. During the stimulation and detection phase, gilts are subjected to daily fence-line and direct exposure to mature boars for the stimulation (primer pheromone effects) and detection (signaling pheromone effect) of pubertal estrus. Daily records of impending estrus during the “priming” phase (progressive vulval changes and behavioral observations of soliciting by the gilt) are recorded. As gilts exhibit their pubertal estrus, confirmed by the back-pressure test, they are weighed and designated to be bred at second or third estrus to achieve target breeding weights. Only gilts with a recorded standing heat (the heat-no-serve event (HNS) event referred to in the N. American industry) are considered to be “select” gilts and eligible to enter the breeding herd. However, if insufficient naturally cycling gilts are available to meet breeding targets after at least 23 days of daily stimulation (an important minimum duration to allow any previously pubertal gilts to exhibit their second heat during the BEAR-based period of recording), eligible “opportunity” gilts (known noncyclic but with an adequate growth rate) can be treated with exogenous gonadotropins (e.g., PG600) and given daily exposure to boars for an additional seven days to confirm an hormonally-induced HNS event. At this point in the GDU program all remaining noncyclic gilts are not eligible to enter the breeding herd and should be culled. 

Patterson et al. [50] reported on the impact of an effective commercially-based GDU program on SLP. Overall, 77.6% of gilts exhibited standing estrus within 35 days of starting boar stimulation at around 190 days of age, consistent with previous results from Amaral Filha et al. [81]. Despite the considerable (and often unexplained) variation in the percentage of gilts naturally cyclic within a group, treatment with PG600 ensured that predictable numbers of high-quality, breeding eligible gilts were available for breeding. Minimal differences in lifetime productivity were reported between gilts with a natural HNS event as compared with those gilts treated with PG600. Most critically, the lifetime retention and performance of gilts entering the breeding herd from this rigorously controlled and monitored GDU/BEAR program exceeded industry benchmarks for SLP. 

Even if the proper facilities are available, the success of a GDU program still depends on the observational ability of the staff involved, regular recording and entry of reproductive events into the farm database, and thorough production record monitoring and analysis [82]. The benefits of data-driven decision making have been demonstrated conclusively across many industries [83] and if GDU-derived data are collected on a regular basis and analyzed effectively, they can be used to make data-driven decisions that will positively affect overall herd performance. Unfortunately, in the case of the replacement gilt, the necessary data is often not collected and/or analyzed. 

## 5. Appropriate Management of Weight, Physiological Maturity, and a Positive Metabolic State at Breeding

Gilts should be stimulated early enough to permit producers to manage gilts to achieve the appropriate weight, estrus number, and metabolic state prior to service after the first detected HNS event [69]. First and second parity litter size has been shown to be predictive of later lifetime performance [2,84,85] and the appropriate management of a gilt at first service is, therefore, important to improve first parity litter size and these lasting effects on lifetime production. The cumulative effective of poor management of the gilt prior to service limits the ability of sows to produce pigs in subsequent parities [10].

### 5.1. Weight

It is recommended that gilts be bred at a target weight of 135 to 150 kg [86,87]. From a biological perspective, the target for service weight is derived from the work of Clowes et al. [88] who reported that a body mass >180 kg after farrowing is protective against the detrimental effects of lean tissue loss during first lactation on subsequent reproductive performance. Thus, if gilts are bred at 135 to 150 kg, and assuming a sow tissue weight gain of 35 to 40 kg during gestation, gilts would be at target weight at farrowing [49,50]. The lower end of the target weight for breeding was also suggested from the empirical study of Williams [86] who reported that gilts weighing less than 135 kg have fewer total pigs born over three parities than gilts weighing over 135 kg. Although Tummaruk and Kesdangsakonwut [68] reported that body weight and growth rate of the gilts was correlated with the number of ovulations and that for every 10 kg increase in body weight an increase of 1.1 corpora lutea was observed, Bortolozzo et al. [7] found no weight-related difference in total born or born alive when gilts were heavier than 130 kg at first service. Furthermore, gilts that were heavier at first service had a decrease in farrowing rate in parity two and those gilts bred at >170 kg were at risk of low retention and locomotion problems over three parities [89]. Heavy gilts at first service, also tend to be heavy at farrowing and have more demands for maintenance over their productive life [7], and heavy gilts during gestation and lactation were reported to achieve less than optimal productivity and feed utilization [89]. Furthermore, compared to slower growing gilts (<700 g/d), gilts with lifetime growth rates from birth to mating >771 g/d had a greater total number of pigs born, but they also had more stillborn pigs and more piglets born weighing less than 1.2 kg. 

Although information on gilt weight at either the onset of boar stimulation, or at the time of pubertal estrus, is a critical step in meeting target gilt breeding weights [49], these records are typically not available across the production industry today [90]. Although a weigh scale is the most accurate way to determine body weight, estimating body weight using a weight tape that is established on the basis allometric growth curves which take advantage of the high correlation between heart girth circumference and body weight, is an objective and simple to manage alternative [90,91].

### 5.2. Estrus at Breeding

Physiological age at breeding (recorded pubertal estrus and number of estrous cycles), rather than chronological age, is an important criterion for determining the time of mating in gilts [49]. Delaying breeding to second estrus has a positive effect on litter size [69,92,93,94] and is generally accepted as common practice in the industry [5]. The increase in litter size is believed to be a consequence of an improvement in ovulation rate after puberty [76,95,96,97,98] and gilts bred at second estrus produced 1.2 more pigs after four litters as compared with gilts bred at first estrus [92]. No improvement in litter size or farrowing rate resulted from delaying breeding beyond second estrus [92,95]. Therefore, breeding beyond second estrus should only be implemented to achieve minimal acceptable breeding weight targets [49], as the accumulation of 21 additional non-productive days may not be offset by an increase in litter size. 

### 5.3. A Positive Metabolic State at Breeding

After the first estrus has been recorded, gilts should be acclimated to stalls or breeding and gestation housing at least 16 d prior to breeding [5]. Studies in the prepubertal gilt [99,100] demonstrated the negative impact of reduced feed intake on the reproductive system and an inhibition of episodic LH secretion within hours of moving gilts from ad libitum to maintenance feed allowances. Thus, the priming mechanisms that will sensitize the ovary to puberty induction stimuli are already affected by the dynamic metabolic state of the gilt. The importance of maintaining high feed intake between first and second estrus to support a maturational increase in ovulation rate from 11.1 to 14.2 ovulations, and some of the endocrine mechanisms involved, were reported by Beltranena et al. [71,98]. Interestingly, more recent studies that manipulated energy intake during the early or late stages of the first estrous cycle did not report detrimental effects of energy restriction on ovulation rate at second estrus (with the average ovulation rate now nearly 18). However, embryonic survival was negatively impacted by restriction in the late but not in the early luteal phase [101,102]. Adequate energy intake during the luteal phase of the first estrous cycle was again confirmed as being crucial to maximizing reproductive performance in gilts, as restricted feed intake had negative effects at the ovarian level and reduced ovulation, and thus potential litter size of gilts bred at second estrus [103]. In this study, feed restriction early in the cycle before breeding was not compensated by high feed intake later in the cycle. Additionally, a starch- (carbohydrate) based energy source was reported to be beneficial for the ovulation rate (16.4 vs. 13.8), numbers of embryos at day 28 (13.4 vs. 11.4), embryo weight (2.0 g vs. 1.7 g) and placental weight (25.3 g vs. 20.8 g) as compared with an oil- (soya bean oil) based energy source [104]. Collectively, these studies provide convincing evidence for maintaining a positive metabolic state in the pre-breeding period in the gilt as another critical step in optimizing herd reproductive performance. Consequently, the practice of moving gilts to individual stalls immediately before breeding will inevitably disrupt normal feed intake and adversely affect the critical priming mechanisms that support ovarian and uterine function and optimize embryo survival and litter size in the gilt [99]. 

## 6. Conclusions

There is substantial evidence supporting successful management of gilts as an absolutely necessary component of herd management and the pivotal starting point for the future fertility and longevity of the breeding herd. Good gilt management starts at birth because litter of origin, lactation management, and the application of early selection strategies are early indicators of future performance and efficiency. Selecting gilts with the greatest potential for lifetime performance is crucial to the productivity of conventional production systems. This can be achieved through the implementation of highly efficient gilt development programs that identify gilts with the greatest reproductive potential, limit entry-to-service intervals, and manage gilts to achieve the appropriate physiological and metabolic state at service. As good gilt management can largely resolve the existing gap between excellent genetic potential and the more modest sow lifetime productivity typically achieved in the industry, an investment in good gilt development programs represents a foundational opportunity for improving the efficiency of the pork production.

## References

[1] Sasaki Y., Koketsu Y. (2008). Sows having high lifetime efficiency and high longevity associated with herd productivity in commercial herds. Livest. Sci..

[2] Koketsu Y., Tani S., Iida R. (2017). Factors for improving reproductive performance of sows and herd productivity in commercial breeding herds. Porc. Health Manag..

[3] Rohrer G.A., Cross A.J., Lents C.A., Miles J.R., Nonneman D.J., Rempel L.A. (2017). 026 Genetic improvement of sow lifetime productivity. J. Anim. Sci..

[4] Kang J.-H., Lee E.-A., Hong K.-C., Kim J.-M. (2018). Regulatory gene network from a genome-wide association study for sow lifetime productivity traits. Anim. Genet..

[5] Kraeling R.R., Webel S.K. (2015). Current strategies for reproductive management of gilts and sows in North America. J. Anim. Sci. Biotechnol..

[6] Ketchem R., Rix M. (2006). National Hog Farmer. https://www.nationalhogfarmer.com/animal-well-being/does-gilt-performance-dictate-farm-success.

[7] Bortolozzo F.P., Bernardi M.L., Kummer R., Wentz I. (2009). Growth, body state and breeding performance in gilts and primiparous sows. Soc. Reprod. Fertil. Suppl..

[8] Patterson J.L., Foxcroft G.R. Troubleshooting reproductive issues. Proceedings of the London Swine Conference.

[9] Nikkila M.T., Stalder K.J., Mote B.E., Rothschild M.F., Gunsett F.C., Johnson A.K., Karricker L.A., Boggess M.V., Serenius T.V. (2013). Genetic associations for gilt growth, compositional, and structural soundness traits with sow longevity and lifetime reproductive performance. J. Anim. Sci..

[10] Patterson J., Foxcroft G. (2019). Gilt management for improved sow lifetime productivity. Advances in Pork Production.

[11] Yuan T., Zhu Y., Shi M., Li T., Li N., Wu G., Bazer F.W., Zang J., Wang F., Wang J. (2015). Within-litter variation in birth weight: Impact of nutritional status in the sow. J. Zhejiang Univ. Sci. B.

[12] Knox R.V. (2019). Physiology and endocrinology symposium: Factors influencing follicle development in gilts and sows and management strategies used to regulate growth for control of estrus and ovulation1. J. Anim. Sci..

[13] Magnabosco D., Pereira Cunha E.C., Bernardi M.L., Wentz I., Bortolozzo F.P. (2015). Impact of the Birth Weight of Landrace × Large White Dam Line Gilts on Mortality, Culling and Growth Performance until Selection for Breeding Herd. Acta Sci. Vet..

[14] Roehe R., Kalm E. (2000). Estimation of genetic and environmental risk factors associated with pre-weaning mortality in piglets using generalized linear mixed models. Anim. Sci..

[15] Almeida F., Dias A.A., Moreira L.P., Fiúza A.T.L., Chiarini-Garcia H. (2017). Ovarian follicle development and genital tract characteristics in different birthweight gilts at 150 days of age. Reprod. Domest. Anim..

[16] Deligeorgis S.G., English P.R., Lodge G.A., Foxcroft G.R. (1985). Interrelationships between growth, gonadotrophin secretion and sexual maturation in gilts reared in different litter sizes. Anim. Prod..

[17] Vallet J.L., Miles J.R., Rempel L.A., Nonneman D.J., Lents C.A. (2015). Relationships between day one piglet serum immunoglobulin immunocrit and subsequent growth, puberty attainment, litter size, and lactation performance. J. Anim. Sci..

[18] Vallet J.L., Calderón-Díaz J.A., Stalder K.J., Phillips C., Cushman R.A., Miles J.R., Rempel L.A., Rohrer G.A., Lents C.A., Freking B.A. (2016). Litter-of-origin trait effects on gilt development. J. Anim. Sci..

[19] Magnabosco D., Bernardi M.L., Wentz I., Cunha E.C.P., Bortolozzo F.P. (2016). Low birth weight affects lifetime productive performance and longevity of female swine. Livest. Sci..

[20] Edwards S.A. (2002). Perinatal mortality in the pig: Environmental or physiological solutions?. Livest. Prod. Sci..

[21] Herpin P., Damon M., Le Dividich J. (2002). Development of thermoregulation and neonatal survival in pigs. Livest. Prod. Sci..

[22] Declerck I., Dewulf J., Sarrazin S., Maes D. (2016). Long-term effects of colostrum intake in piglet mortality and performance. J. Anim. Sci..

[23] Rutherford K.M.D., Baxter E.M., D’Eath R.B., Turner S.P., Arnott G., Roehe R., Ask B., Sandøe P., Moustsen V.A., Thorup F. (2013). The welfare implications of large litter size in the domestic pig I: Biological factors. Anim. Welf..

[24] Wiegert J.G., Garrison C., Knauer M.T. (2017). 068 Characterization of birth weight and colostrum intake on piglet survival and piglet quality. J. Anim. Sci..

[25] Bartol F.F., Wiley A.A., Miller D.J., Silva A.J., Roberts K.E., Davolt M.L.P., Chen J.C., Frankshun A.-L., Camp M.E., Rahman K.M. (2013). Lactation biology symposium: Lactocrine signaling and developmental programming. J. Anim. Sci..

[26] Ferrari C.V., Sbardella P.E., Bernardi M.L., Coutinho M.L., Vaz I.S., Wentz I., Bortolozzo F.P. (2014). Effect of birth weight and colostrum intake on mortality and performance of piglets after cross-fostering in sows of different parities. Prev. Vet. Med..

[27] Nelson R.E., Robinson O.W. (1976). Effects of Postnatal Maternal Environment on Reproduction of Gilts. J. Anim. Sci..

[28] Flowers W.L. Effect of Neonatal Litter Size and Early Puberty Stimulation on Sow Longevity and Reproductive Performance. https://www.pork.org/research/effect-of-neonatal-litter-size-and-early-puberty-stimulation-on-sow-longevity-and-reproductive-performance/.

[29] Flowers W.L. (2018). Personal Communication.

[30] Foxcroft G.R., Dixon W.T., Dyck M.K., Novak S., Harding J.C.S., Almeida F.C.R.L. (2009). Prenatal programming of postnatal development in the pig. Soc. Reprod. Fertil. Suppl..

[31] Smit M.N., Spencer J.D., Almeida F.R.C.L., Patterson J.L., Chiarini-Garcia H., Dyck M.K., Foxcroft G.R. (2013). Consequences of a low litter birth weight phenotype for postnatal lean growth performance and neonatal testicular morphology in the pig. Anim. Int. J. Anim. Biosci..

[32] Da Silva C.L.A., Mulder H.A., Broekhuijse M.L.W.J., Kemp B., Soede N.M., Knol E.F. (2018). Relationship Between the Estimated Breeding Values for Litter Traits at Birth and Ovarian and Embryonic Traits and Their Additive Genetic Variance in Gilts at 35 Days of Pregnancy. Front. Genet..

[33] Patterson J., Foxcroft G., Holden N., Allerson M., Hanson A., Triemert E., Bruner L., Pinilla J.C. (2018). A Low Litter Birth Weight Phenotype Reduces the Retention Rate of Potential Replacement *Gilts*. J. Anim. Sci..

[34] Seyfang J., Kirkwood R.N., Tilbrook A.J., Ralph C.R. (2018). The sex ratio of a gilt’s birth litter can affect her fitness as a breeding female. Anim. Prod. Sci..

[35] Rekiel A., Więcek J., Wojtasik M., Ptak J., Blicharski T., Mroczko L. (2012). Effect of Sex Ratio in the Litter in Which Polish Large White and Polish Landrace Sows were Born on the Number of Piglets Born and Reared. Ann. Anim. Sci..

[36] Seyfang J., Ralph C.R., Hebart M.L., Tilbrook A.J., Kirkwood R.N. (2018). Anogenital distance reflects the sex ratio of a gilt’s birth litter and predicts her reproductive success1. J. Anim. Sci..

[37] Lamberson W.R., Blair R.M., Rohde Parfet K.A., Day B.N., Johnson R.K. (1988). Effect of Sex Ratio of the Birth Litter on Subsequent Reproductive Performance of Gilts. J. Anim. Sci..

[38] Drickamer L.C., Arthur R.D., Rosenthal T.L. (1997). Conception failure in swine: Importance of the sex ratio of a female’s birth litter and tests of other factors. J. Anim. Sci..

[39] Drickamer L.C., Rosenthal T.L., Arthur R.D. (1999). Factors affecting the number of teats in pigs. Reproduction.

[40] Seyfang J., Plush K.J., Kirkwood R.N., Tilbrook A.J., Ralph C.R. (2018). The sex ratio of a litter affects the behaviour of its female pigs until at least 16 weeks of age. Appl. Anim. Behav. Sci..

[41] Fitzgerald R. (2009). An Evaluation or Practices to Improve Sow Productive Lifetime and Producer Profitability. Ph.D. Thesis.

[42] Bergman P., Gröhn Y.T., Rajala-Schultz P., Virtala A.-M., Oliviero C., Peltoniemi O., Heinonen M. (2018). Sow removal in commercial herds: Patterns and animal level factors in Finland. Prev. Vet. Med..

[43] Stalder K.J., Lacy R.C., Cross T.L., Conatser G.E. (2003). Financial impact of average parity of culled females in a breed-to-wean swine operation using replacement gilt net present value analysis. J. Swine Health Prod..

[44] Engblom L., Díaz J.A.C., Nikkilä M., Gray K., Harms P., Fix J., Tsuruta S., Mabry J., Stalder K. (2016). Genetic analysis of sow longevity and sow lifetime reproductive traits using censored data. J. Anim. Breed. Genet..

[45] Gruhot T.R., Díaz J.A.C., Baas T.J., Dhuyvetter K.C., Schulz L.L., Stalder K.J. (2017). An economic analysis of sow retention in a United States breed-to-wean system. J. Swine Health Prod..

[46] Li Q., Yuan X., Chen Z., Zhang A., Zhang Z., Zhang H., Li J. (2018). Heritability estimates and effect on lifetime reproductive performance of age at puberty in sows. Anim. Reprod. Sci..

[47] Roongsitthichai A., Cheuchuchart P., Chatwijitkul S., Chantarothai O., Tummaruk P. (2013). Influence of age at first estrus, body weight, and average daily gain of replacement gilts on their subsequent reproductive performance as sows. Livest. Sci..

[48] Engblom L., Lundeheim N., Dalin A.-M., Andersson K. (2007). Sow removal in Swedish commercial herds. Livest. Sci..

[49] Foxcroft G., Patterson J. Optimizing breeding management in a competitive world: Gilt and sow aspects. Proceedings of the AASV 41st Annual Meeting.

[50] Patterson J., Triemert E., Gustafson B., Werner T., Holden N., Pinilla J.C., Foxcroft G. (2016). Validation of the use of exogenous gonadotropins (PG600) to increase the efficiency of gilt development programs without affecting lifetime productivity in the breeding herd. J. Anim. Sci..

[51] Serenius T., Stalder K.J. (2007). Length of productive life of crossbred sows is affected by farm management, leg conformation, sow’s own prolificacy, sow’s origin parity and genetics. Anim. Int. J. Anim. Biosci..

[52] Wijesena H.R., Lents C.A., Riethoven J.J., Trenhaile-Grannemann M.D., Thorson J.F., Keel B.N., Miller P.S., Spangler M.L., Kachman S.D., Ciobanu D.C. (2017). Genomics Symposium: Using genomic approaches to uncover sources of variation in age at puberty and reproductive longevity in sows. J. Anim. Sci..

[53] Knauer M.T., Cassady J.P., Newcom D.W., See M.T. (2011). Phenotypic and genetic correlations between gilt estrus, puberty, growth, composition, and structural conformation traits with first-litter reproductive measures. J. Anim. Sci..

[54] Sterning M., Rydhmer L., Eliasson-Selling L. (1998). Relationships between age at puberty and interval from weaning to estrus and between estrus signs at puberty and after the first weaning in pigs. J. Anim. Sci..

[55] Patterson J.L., Beltranena E., Foxcroft G.R. (2010). The effect of gilt age at first estrus and breeding on third estrus on sow body weight changes and long-term reproductive performance. J. Anim. Sci..

[56] Tart J.K., Johnson R.K., Bundy J.W., Ferdinand N.N., McKnite A.M., Wood J.R., Miller P.S., Rothschild M.F., Spangler M.L., Garrick D.J. (2013). Genome-wide prediction of age at puberty and reproductive longevity in sows. Anim. Genet..

[57] Knauer M., Stalder K.J., Serenius T., Baas T.J., Berger P.J., Karriker L., Goodwin R.N., Johnson R.K., Mabry J.W., Miller R.K. (2010). Factors associated with sow stayability in 6 genotypes. J. Anim. Sci..

[58] Saito H., Sasaki Y., Koketsu Y. (2011). Associations between Age of Gilts at First Mating and Lifetime Performance or Culling Risk in Commercial Herds. J. Vet. Med. Sci..

[59] Tani S., Koketsu Y. (2016). Factors for Culling Risk due to Pregnancy Failure in Breeding-Female Pigs. J. Agric. Sci..

[60] Schukken Y.H., Buurman J., Huirne R.B., Willemse A.H., Vernooy J.C., van den Broek J., Verheijden J.H. (1994). Evaluation of optimal age at first conception in gilts from data collected in commercial swine herds. J. Anim. Sci..

[61] Koketsu Y., Takahashi H., Akachi K. (1999). Longevity, Lifetime Pig Production and Productivity, and Age at First Conception in a Cohort of Gilts Observed over Six Years on Commercial Farms. J. Vet. Med. Sci..

[62] Magnabosco D., Cunha E.C.P., Bernardi M.L., Wentz I., Bortolozzo F.P. (2014). Effects of age and growth rate at onset of boar exposure on oestrus manifestation and first farrowing performance of Landrace×large white gilts. Livest. Sci..

[63] Calderón Díaz J.A., Vallet J.L., Lents C.A., Nonneman D.J., Miles J.R., Wright E.C., Rempel L.A., Cushman R.A., Freking B.A., Rohrer G.A. (2015). Age at puberty, ovulation rate, and uterine length of developing gilts fed two lysine and three metabolizable energy concentrations from 100 to 260 d of age. J. Anim. Sci..

[64] Van Wettere W.H.E.J., Revell D.K., Mitchell M., Hughes P.E. (2006). Increasing the age of gilts at first boar contact improves the timing and synchrony of the pubertal response but does not affect potential litter size. Anim. Reprod. Sci..

[65] Knox R.V., Rodriguez Zas S.L., Sloter N.L., McNamara K.A., Gall T.J., Levis D.G., Safranski T.J., Singleton W.L. (2013). An analysis of survey data by size of the breeding herd for the reproductive management practices of North American sow farms. J. Anim. Sci..

[66] Stančić B., Gagrčin M., Grafenau P.S., Grafenau P.J., Stančić I., Kubovičová E., Pivko J. (2007). Morphological examination of ovaries in gilts with not detected standing oestrus up to 240 days of age and later. Slovak J. Anim. Sci..

[67] Stancic I., Stancic B., Bozic A., Anderson R., Harvey R., Gvozdic D. (2011). Ovarian activity and uterus organometry in delayed puberty gilts. Theriogenology.

[68] Tummaruk P., Kesdangsakonwut S. (2015). Number of ovulations in culled Landrace × Yorkshire gilts in the tropics associated with age, body weight and growth rate. J. Vet. Med. Sci..

[69] Levis D. Housing and management aspects influencing gilt development and longevity: A review. Proceedings of the 2000 Allen D. Leman Conference.

[70] Kirkwood R.N., Aherne F.X. (1985). Energy Intake, Body Composition and Reproductive Performance of the Gilt. J. Anim. Sci..

[71] Beltranena E., Aherne F.X., Foxcroft G.R., Kirkwood R.N. (1991). Effects of pre- and postpubertal feeding on production traits at first and second estrus in gilts. J. Anim. Sci..

[72] Kummer R., Bernardi M.L., Schenkel A.C., Filha W.A., Wentz I., Bortolozzo F.P. (2009). Reproductive Performance of Gilts with Similar Age but with Different Growth Rate at the Onset of Puberty Stimulation. Reprod. Domest. Anim..

[73] Hughes P.E., Pearce G.P., Paterson A.M. (1990). Mechanisms mediating the stimulatory effects of the boar on gilt reproduction. J. Reprod. Fertil. Suppl..

[74] Kirkwood R.N., Forbes J.M., Hughes P.E. (1981). Influence of boar contact on attainment of puberty in gilts after removal of the olfactory bulbs. J. Reprod. Fertil..

[75] Beltranena E., Patterson J., Foxcroft G. Designing effective boar stimulation systems as a critical feature of the gilt development unit. Proceedings of the Allen D. Leman Pre-Conference Reproduction Workshop, Effective Management of Replacement Gilts.

[76] Knox R., Daniel A., Patterson J., Arend L., Foxcroft G. Effects of birth traits, physical or fenceline boar exposure and group size on pubertal measures and lifetime fertility of replacement gilts. Proceedings of the Billy Day Symposium.

[77] Patterson J.L., Willis H.J., Kirkwood R.N., Foxcroft G.R. (2002). Impact of boar exposure on puberty attainment and breeding outcomes in gilts. Theriogenology.

[78] Rekwot P.I., Ogwu D., Oyedipe E.O., Sekoni V.O. (2001). The role of pheromones and biostimulation in animal reproduction. Anim. Reprod. Sci..

[79] Zimmerman D., McGargill T., Rohda N., Anderson M. (1997). Boar Libido Affects Pubertal Development of Gilts. Neb. Swine Rep..

[80] Paterson A.M., Hughes P.E., Pearce G.P. (1989). The effect of season, frequency and duration of contact with boars on the attainment of puberty in gilts. Anim. Reprod. Sci..

[81] Amaral Filha W.S., Bernardi M.L., Wentz I., Bortolozzo F.P. (2009). Growth rate and age at boar exposure as factors influencing gilt puberty. Livest. Sci..

[82] Evans L., Britt J., Kirkbride C., Levis D. (2006). Troubleshooting Swine Reproduction Failure. Pork Information Gateway. http://porkgateway.org/wp-content/uploads/2015/07/troubleshooting-swine-reproduction-failure1.pdf.

[83] Provost F., Fawcett T. (2013). Data Science and its Relationship to Big Data and Data-Driven Decision Making. Big Data.

[84] Gruhot T.R., Calderón Díaz J.A., Baas T.J., Stalder K.J. (2017). Using first and second parity number born alive information to estimate later reproductive performance in sows. Livest. Sci..

[85] Iida R., Koketsu Y. (2015). Number of pigs born alive in parity 1 sows associated with lifetime performance and removal hazard in high- or low-performing herds in Japan. Prev. Vet. Med..

[86] Williams N.H., Patterson J.L., Foxcroft G.R. (2005). Non-negotiables in gilt development. Adv. Pork Prod..

[87] Kim J.S., Yang X., Baidoo S.K. (2016). Relationship between Body Weight of Primiparous Sows during Late Gestation and Subsequent Reproductive Efficiency over Six Parities. Asian-Australas. J. Anim. Sci..

[88] Clowes E.J., Aherne F.X., Schaefer A.L., Foxcroft G.R., Baracos V.E. (2003). Parturition body size and body protein loss during lactation influence performance during lactation and ovarian function at weaning in first parity sows. J. Anim. Sci..

[89] Filha W.S.A., Bernardi M.L., Wentz I., Bortolozzo F.P. (2010). Reproductive performance of gilts according to growth rate and backfat thickness at mating. Anim. Reprod. Sci..

[90] Al Ard Khanji M.S., Llorente C., Falceto M.V., Bonastre C., Mitjana O., Tejedor M.T. (2018). Using body measurements to estimate body weight in gilts. Can. J. Anim. Sci..

[91] Pasternak J., Patterson J., Cameron A., Dyck M., Foxcroft G. (2008). The Use of Allometric Relationships to Estimate Gilt Body Weight. Adv. Pork Prod..

[92] Young L.G., King G.J., Walton J.S., McMillan I., Klevorick M. (1990). Reproductive Performance over Four Parities of Gilts Stimulated to Early Estrus and Mated at First, Second or Third Observed Estrus. Can. J. Anim. Sci..

[93] MacPherson R.M., Hovell F.D.D., Jones A.S. (1977). Performance of sows first mated at puberty or second or third oestrus, and carcass assessment of once-bred gilts. Anim. Sci..

[94] Walker N., Kilpatrick D.J., Courtney D.J. (1989). The Effect of Conception in Gilts at Puberty or Second Oestrus on Reproductive Performance over Two Parities. Ir. J. Agric. Res..

[95] Grigoriadis D.F., Edwards S.A., English P.R., Davidson F. (2001). The effect of oestrous cycle number, at constant age, on gilt reproduction in a dynamic service system. Anim. Sci..

[96] Aherne F.X., Williams I.H., Head R.H. Nutrition—Reproduction interactions in swine. Proceedings of the Recent Advances in Animal Nutrition Conference.

[97] Gaughan J.B., Cameron R.D., Dryden G.M., Young B.A. (1997). Effect of body composition at selection on reproductive development in large white gilts. J. Anim. Sci..

[98] Beltranena E., Foxcroft G.R., Aherne F.X., Kirkwood R.N. (1991). Endocrinology of nutritional flushing in gilts. Can. J. Anim. Sci..

[99] Booth P.J., Cosgrove J.R., Foxcroft G.R. (1996). Endocrine and metabolic responses to realimentation in feed-restricted prepubertal gilts: Associations among gonadotropins, metabolic hormones, glucose, and uteroovarian development. J. Anim. Sci..

[100] Booth P.J., Craigon J., Foxcroft G.R. (1994). Nutritional manipulation of growth and metabolic and reproductive status in prepubertal gilts. J. Anim. Sci..

[101] Almeida F.R.C.L., Kirkwood R.N., Aherne F.X., Foxcroft G.R. (2000). Consequences of different patterns of feed intake during the estrous cycle in gilts on subsequent fertility. J. Anim. Sci..

[102] Almeida F.R.C.L., Mao J., Novak S., Cosgrove J.R., Foxcroft G.R. (2001). Effects of different patterns of feed restriction and insulin treatment during the luteal phase on reproductive, metabolic, and endocrine parameters in cyclic gilts. J. Anim. Sci..

[103] Chen T.Y., Stott P., Athorn R.Z., Bouwman E.G., Langendijk P. (2012). Undernutrition during early follicle development has irreversible effects on ovulation rate and embryos. Reprod. Fertil. Dev..

[104] Almeida F.R.C.L., Machado G.S., Borges A.L.C.C., Rosa B.O., Fontes D.O. (2014). Consequences of different dietary energy sources during follicular development on subsequent fertility of cyclic gilts. Animal.

